# RBT-1 reduces blood product utilization in patients undergoing nonemergency coronary artery bypass grafting and/or valve surgery

**DOI:** 10.1016/j.xjon.2024.06.019

**Published:** 2024-07-01

**Authors:** Charles A. Mack, Michael Jessen, Andre Lamy, Ashish K. Khanna, Kevin Lobdell, Rakesh Arora, Jeannette Rodriguez, Stacey Ruiz, Bhupinder Singh

**Affiliations:** a Department of Cardiothoracic Surgery, Weill Cornell Medicine, New York, NY; b Department of Cardiovascular and Thoracic Surgery, University of Texas Southwestern, Dallas, Tex; c Department of Perioperative Medicine and Surgery, Population Health Research Institute, Hamilton, Ontario, Canada; d Perioperative Outcomes and Informatics Collaborative, Section on Critical Care Medicine, Department of Anesthesiology, Wake Forest University School of Medicine, Atrium Health Wake Forest Baptist Medical Center, Winston-Salem, NC; e Outcomes Research Consortium, Cleveland, Ohio; f Sanger Heart and Vascular Institute, Atrium Health, Charlotte, NC; g Division of Cardiac Surgery, Department of Surgery, Harrington Heart and Vascular Institute, University Hospitals–Cleveland Medical Center, Case Western Reserve University, Cleveland, Ohio; h Department of Drug Development, Renibus Therapeutics, Inc, Southlake, Tex; i Medical Office, Renibus Therapeutics, Inc, Southlake, Tex

**Figure:**
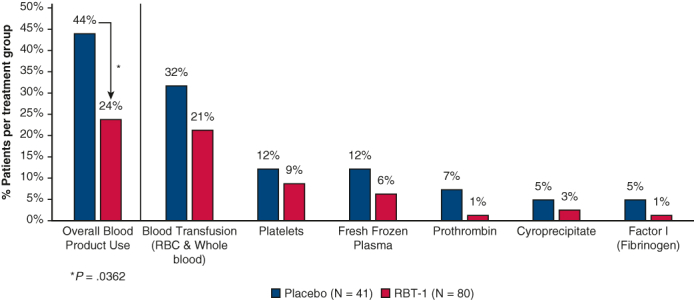
Influence of RBT-1 versus placebo on blood product utilization in cardiac surgery patients.

**Central Message**

In this Phase 2 study, patients undergoing CABG and/or valve surgery treated with RBT-1 required less blood transfusion/blood product use compared with placebo.

Perioperative anemia occurs in up to 54% of patients undergoing cardiac surgery and is associated with decreased oxygen delivery, which can contribute to end-organ dysfunction and adversely affect post-operative outcomes.^1^ Factors that contribute to perioperative anemia include blood loss, hemodilution, and hemolysis. Strategies to reduce or address perioperative anemia include intraoperative antifibrinolytics, cell salvage, intraoperative retrograde autologous prime and intraoperative autologous donation.^2^^,^^3^ Administration of blood products is associated with adverse clinical outcomes and may not mitigate the risks of anemia.^1^^,^^4^

RBT-1, a combination of stannic protoporfin (SnPP) and iron sucrose (FeS), is a cytoprotective preconditioning agent that activates antioxidant, anti-inflammatory, and iron-scavenging pathways. In a Phase 2 study, it was shown to upregulate a preconditioning biomarker response and decrease postoperative complications after coronary artery bypass graft (CABG) and/or valve surgery on cardiopulmonary bypass (CPB).^5^ The purpose of the current analysis was to evaluate whether the pleiotropic effects of RBT-1 would reduce the overall need for blood products. Because complications requiring blood product use may also prolong time on ventilator and in the intensive care unit (ICU), these outcomes were also evaluated as exploratory outcomes.

## Materials and Methods

### Study Design

The underlying study was a Phase 2, multicenter, randomized, double-blind, placebo-controlled trial.^5^ Patients were randomized 1:1:1 to receive low dose RBT-1 (45 mg SnPP/240 mg FeS), high dose RBT-1 (90 mg SnPP/240 mg FeS), or placebo, as a single intravenous infusion 24 to 48 hours before surgery. Infusion was administered on an inpatient or outpatient basis, depending on the patient’s status. Patients were followed through 90 days postsurgery. Institutional review board approval was obtained (project No.: Pro00047629; November 6, 2020), and each patient provided informed consent for publication.

### Study Participants

As reported previously by Lamy and colleagues,^5^ eligible patients were those aged 18 years or older who were scheduled to undergo nonemergency CABG and/or valve surgery on CPB. Key exclusion criteria included acute organ dysfunction (eg, acute kidney injury and acute livery injury), estimated glomerular filtration rate ≤20 mL/minute/1.73 m^2^ or need for dialysis, intraoperative circulatory arrest or deep hypothermia, requirement for inotropes or vasopressors or other mechanical devices prior to surgery, active infection, and serum ferritin >500 ng/mL.

### Study End Points

In this exploratory analysis, the effect of RBT-1 on blood product usage was assessed. Blood product use was recorded and included whole blood, red blood cells, platelets, fresh frozen plasma, prothrombin, cryoprecipitate, and Factor I (fibrinogen). Hemoglobin values were determined at a central laboratory. Predefined thresholds were not instituted for blood transfusion or use of other blood products. The need for blood products was assessed by each investigator according to the standard of care at each study center. A transfusion protocol was not used for this study; however, site level randomization was employed to account for differences in standard of care at each institution. Days on ventilator and in the ICU were also recorded.

### Sample Size Calculation

This sample size for this study was estimated to be N = 126 based on >80% power to achieve a 2-sided ***α*** = 0.05 for the primary end point (upregulation of a preconditioning biomarker response) and allowance of safety assessments as needed for proof-of-concept studies, as previously reported.^5^

### Statistical Analysis

The modified intention-to-treat population, which consisted of all patients who received drug and underwent surgery on time (ie, 24-48 hours postinfusion of study drug) was evaluated for clinical outcomes.^5^ Categorical variables were summarized using proportions, and continuous variables were summarized using mean ± SD. Given the lack of statistically significant differences between the low dose and high dose RBT-1 groups, all patients randomized to either dose of RBT-1 were combined into one group for comparison with those who received placebo. All between-group comparisons were assessed using a 2-sided ***α*** = 0.05. Further statistical details can be found in Lamy and colleagues.^5^

## Results

Of the 152 patients enrolled in this study across the United States, Canada, and Australia, 121 comprised the modified intention-to-treat population.^5^ Baseline characteristics were similar across treatment groups, although those randomized to RBT-1 had a slightly higher risk for post-op complications (Table 1). Mean platelet levels at baseline were numerically lower in the RBT-1 treatment group (226.3 × 10^9^/L) than in the placebo group (233.5 × 10^9^/L). The majority of the surgeries performed in each treatment group were CABG, with more valve surgeries performed in the RBT-1 group and more combined CABG/valve surgeries performed in the placebo group. Only 2 patients (both treated with RBT-1) required re-exploration. Additionally, the duration of surgery and time on CPB were similar in both treatment groups. The duration of surgery was 4.66 and 5.00 hours in the placebo and RBT-1 groups, respectively, and the duration of CPB times was 1.95 and 1.97 hours in the placebo and RBT-1 groups, respectively.

**Table 1:** Baseline characteristics

Characteristic	Placebo (n = 41)	RBT-1 (n = 80)
Age (y)	65.4 ± 11.0	65.6 ± 9.8
Male	30 (73.2)	60 (75.0)
Race		
American Indian	0 (0.0)	1 (1.3)
Black	2 (4.9)	5 (6.3)
Asian	1 (2.4)	3 (3.8)
White	38 (92.7)	69 (86.3)
Other	0 (0.0)	2 (2.5)
Weight (kg)	89.1 ± 18.5	94.2 ± 20.6
Body mass index	29.7 ± 5.5	31.5 ± 6.6
Age ≥65 y	23 (56.1)	48 (60.0)
Diabetes mellitus requiring insulin	3 (7.3)	14 (17.5)
Congestive heart failure	6 (14.6)	12 (15.0)
Heart failure (NYHA III/IV) within 1 year before surgery	2 (4.9)	8 (10.0)
Previous cardiac surgery with sternotomy	0 (0.0)	2 (2.5)
LVEF ≤35%	2 (4.9)	9 (11.3)
eGFR ≥20 to <60 mL/min/1.73 m^2^	6 (14.6)	26 (32.5)
Platelet levels, mean ± SD	233.5 ± 60.5 × 10^9^/L	226.3 ± 60.3 × 10^9^/L
Time on CPB (h)	1.95 ± 1.05	1.97 ± 1.01
Surgery type		
CABG	20 (48.8)	44 (55.0)
Valve	7 (17.1)	22 (27.5)
Combined CABG/valve	14 (34.1)	14 (17.5)

Values are presented as mean ± SD or n (%). *RBT-1*, Combination of stannic protoporfin and iron sucrose; *NYHA*, New York Heart Association; *LVEF*, left ventricular ejection fraction; *eGFR*, estimated glomerular filtration rate; *CPB*, cardiopulmonary bypass; *CABG*, coronary artery bypass graft.

The incidence of blood product utilization was 43.9% (18 of 41 patients) in the placebo group and 23.8% (19 out of 80 patients) in the RBT-1 group, demonstrating a significant reduction with RBT-1 (−46% risk reduction; *P* = .0362) (Table 2). Hemoglobin levels were similar across treatment groups through 90 days postsurgery (Table 2), despite less use of blood products in patients treated with RBT-1. Utilization of individual blood product components was also reduced, with the greatest influence on blood transfusions (red blood cells [RBCs]/whole blood) in response to RBT-1 (21.3% [17 out of 80 patients]) compared with placebo (31.7% [13 out of 41 patients]). These data are consistent with the clinically meaningful reduction in time on ventilator in patients receiving RBT-1 versus placebo (1.00-day difference; 41% risk reduction) and statistically significant reduction in ICU days in patients receiving RBT-1 versus placebo (2.71-day difference; 45% risk reduction; *P* = .0243).

**Table 2:** Hemoglobin levels, utilization of blood products, and key clinical outcomes

Study endpoint	Placebo (n = 41)	RBT-1 (n = 80)
Hemoglobin (g/dL)		
Baseline	13.8 ± 1.477	13.8 ± 1.533
Day 7 or discharge	9.8 ± 1.717	9.8 ± 1.325
Day 30	11.7 ± 1.339	11.8 ± 1.213
Day 60	12.3 ± 1.123	12.6 ± 1.267
Day 90	12.9 ± 1.179	13.3 ± 1.252
Composition of blood products∗		
Overall	43.9	23.8†
Blood transfusion, RBCs and whole blood	31.7	21.3
Platelets	12.2	8.8
Blood plasma	12.2	6.3
Prothrombin	7.3	1.3
Cryoprecipitate	4.9	2.5
Fibrinogen	4.9	1.3
Time on ventilator and in intensive care		
Ventilator (d)	2.44	1.44
Intensive care unit (d)	6.00	3.29‡

Values are presented as mean ± SD or %, unless otherwise noted. *RBT-1*, Combination of stannic protoporfin and iron sucrose; *RBC*, red blood cell.

∗ The sum of the individual blood products does not add up to the overall percentage because some patients received more than 1 type of blood product.

† *P* = .0362.

‡ *P* = .0243.

Given that a higher percentage of patients in the placebo group underwent combined CABG/valve surgery, we assessed whether this imbalance may have contributed to the differences in blood product usage between groups. Blood product usage remained lower in RBT-1 group (50% [7 out of 14 patients]) versus placebo group (71% [10 of 14 patients]) for this subset of patients.

**Figure 1:**
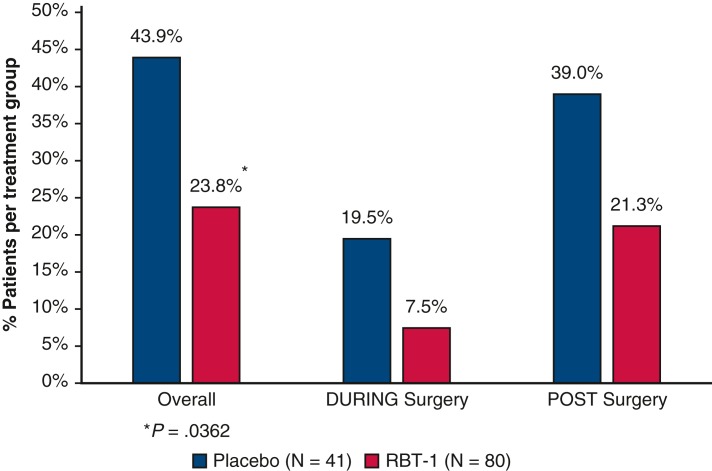
The use of blood products overall, during surgery, and after surgery are shown by treatment group. The sum of patients requiring blood product usage during surgery and postsurgery does not add up to the overall patients requiring blood products because some patients received blood products both during and postsurgery. *RBT-1*, Combination of stannic protoporfin and iron sucrose.

Blood product use was also evaluated separately during and following surgery (Figure 1). During surgery, 7.5% of RBT-1–treated patients required blood product use compared with 19.5% of placebo-treated patients; following surgery, blood product use was 21.3% and 39.0%, respectively.

## Discussion

Perioperative anemia, which requires blood/blood product transfusions to increase blood oxygen carrying capacity and raise hematocrit, is associated with adverse long-term immunologic effects, risk of viral transmission and acute transfusion reactions, and worse postoperative outcomes overall.^1^

RBT-1 treatment reduced the need for blood transfusion and use of blood products in patients undergoing CABG and/or heart valve surgery, providing a potential therapeutic approach to reducing the transfusion of RBCs/whole blood and use of blood products associated with cardiac surgery. Additionally, the reduction in blood product use may contribute to improved clinical outcomes, as observed in reduced time on ventilator and time in ICU.

The mechanism by which RBT-1 may reduce the need for blood products, and specifically blood transfusions, is likely multifactorial. The drug contains iron sucrose, which provides an exogenous source of iron perioperatively.^6^ Related increases in ferritin levels may help process free iron released during CPB and, hence, aid in maintaining adequate hematopoiesis.^5^^,^^7^ In addition, broad antioxidant and anti-inflammatory effects of the drug mediated via heme oxygenase-1 and interleukin-10 pathways may promote cytoresistance to surgical stress, thus resulting in cellular preservation, such as that of RBCs (including reduced RBC rigidity and fragility), platelets, and endothelial cells.^5^^,^^8^, ^9^, ^10^

### Study Limitations

A limitation of this study is the lack of a predefined protocol for blood product usage; therefore, the rates of blood product usage may have been influenced by differences in institutional practices and individual discretion. Additionally, there was not a set protocol for cessation of antiplatelet agents before surgery, which may have confounded the need for blood products. The total volume of blood loss was not measured in this study; therefore, variations in the practice of stopping antiplatelet agents could have influenced the need for blood products. Randomization at the site level was implemented to reduce the influence of institutional practice differences on outcomes.

## Conclusions

Given that worse postoperative outcomes are associated with perioperative anemia,^1^ continued investigation of RBT-1 is warranted. A Phase 3 study (PROTECT [Phase 3, Randomized, Double-Blind, Placebo-Controlled Study to Evaluate the Effect of RBT-1 on Reducing the Risk of Post-Operative Complications in Subjects Undergoing Cardiac Surgery]; NCT06021457) will further evaluate these findings.

### Webcast

You can watch a Webcast of this AATS meeting presentation by going to: https://www.aats.org/resources/rbt-1-reduces-incidence-of-blo-7357.

**Figure:**
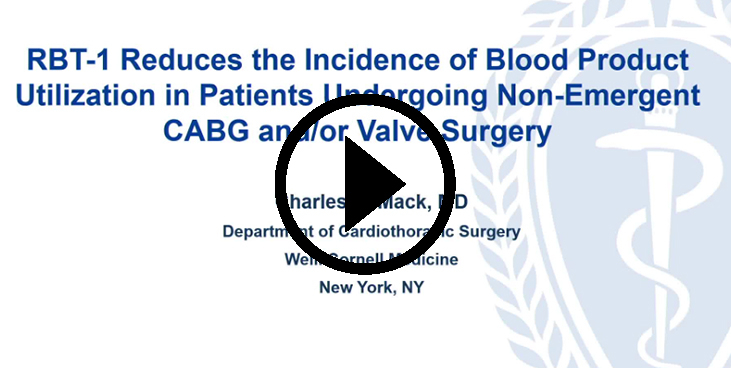

## Conflict of Interest Statement

AKK is consultant for Renibus Therapeutics and consults for Medtronic, Edwards Lifesciences, Philips Research North America, Baxter, GE Healthcare, Potrero Medical, Retia Medical, and Caretaker Medical outside of this work. Drs Rodriguez, Ruiz, and Singh are employees of Renibus Therapeutics Inc. All other authors reported no conflicts of interest.

The *Journal* policy requires editors and reviewers to disclose conflicts of interest and to decline handling or reviewing manuscripts for which they may have a conflict of interest. The editors and reviewers of this article have no conflicts of interest.
